# Retraction: Differential expression of sirtuins in the aging rat brain

**DOI:** 10.3389/fncel.2026.1868495

**Published:** 2026-05-04

**Authors:** Frontiers Editorial Office

The journal retracts the article cited above.

Following publication, concerns were raised regarding the integrity of the images in the published figures, and data presented in the article. The authors' institution conducted an investigation and found that the concerns were valid, therefore the editors no longer have confidence in the findings presented in the article.

This retraction was approved by the Chief Editors of Frontiers in Cellular Neuroscience and the Chief Executive Editor of Frontiers. The authors received a communication regarding the retraction and had a chance to respond. This communication has been recorded by the publisher.

Frontiers would like to thank the concerned reader who contacted us regarding the published article, Elisabeth Bik, and anonymous PubPeer users for bringing these issues to our attention.

